# Effectiveness of a telephone-based intervention for smoking cessation in patients with severe mental disorders: study protocol for a randomized controlled trial

**DOI:** 10.1186/s13063-018-3106-5

**Published:** 2019-01-11

**Authors:** Montse Ballbè, Cristina Martínez, Ariadna Feliu, Núria Torres, Gemma Nieva, Cristina Pinet, Antònia Raich, Sílvia Mondon, Pablo Barrio, Rosa Hernández-Ribas, Jordi Vicens, Sílvia Costa, Jordi Vilaplana, Laura Alaustre, Eva Vilalta, Roser Blanch, Susana Subirà, Eugeni Bruguera, Josep Maria Suelves, Joseph Guydish, Esteve Fernández

**Affiliations:** 1 Tobacco Control Unit, Cancer Control and Prevention Program, Institut Català d’Oncologia-ICO, Av. Granvia de L’Hospitalet 199-203, L’Hospitalet de Llobregat, 08908 Barcelona, Spain; 2 Cancer Control and Prevention Group, Institut d’Investigació Biomèdica de Bellvitge-IDIBELL, Av. Granvia de L’Hospitalet 199-203, L’Hospitalet de Llobregat, 08908 Barcelona, Spain; 3 Addictions Unit, Institute of Neurosciences, Hospital Clínic de Barcelona, C. Villarroel 170, 08036 Barcelona, Spain; 4 Medicine and Health Sciences School, Universitat Internacional de Catalunya, C. Josep Trueta s/n, 08915 Sant Cugat del Valles, Barcelona, Spain; 5 Department of Clinical Sciences, School of Medicine, Universitat de Barcelona, C. Feixa Llarga s/n, L’Hospitalet del Llobregat, 08907 Barcelona, Spain; 6 061 CatSalut Respon, Sistema d’Emergències Mèdiques, C. Pablo Iglesias 115, L’Hospitalet de Llobregat, 08908 Barcelona, Spain; 7 Smoking Cessation Unit, Addictive Behaviors Unit, Psychiatry Department, Hospital Universitari Vall d’Hebron, Vall d’Hebron Institute of Research, CIBERSAM, Universitat Autònoma de Barcelona, Passeig de la Vall d’Hebron 119-129, 08035 Barcelona, Spain; 8 Addictive Behaviors Unit, Psychiatry Department, Hospital de la Santa Creu i Sant Pau, C. San Antoni Ma Claret 167, 08025 Barcelona, Spain; 9 Mental Health Department, Althaia Xarxa Assistencial Universitària, C. Dr. Llatjós s/n, Manresa, 08243 Barcelona, Spain; 10 Alcohol Program, Psychiatry Department, Hospital Universitari de Bellvitge, Institut Català d’Oncologia, IDIBELL, CIBERSAM, Feixa Llarga s/n, L’Hospitalet de Llobregat, 08907 Barcelona, Spain; 11 Psychiatry Department, Hestia Duran i Reynals, Av. Granvia de L’Hospitalet 199-203, L’Hospitalet de Llobregat, 08908 Barcelona, Spain; 12 Institut d’Investigació Biomèdica Sant Pau, C. San Antoni Mª Claret 167, 08025 Barcelona, Spain; 13 Lleida Institute for Biomedical Research Dr. Pifarré Foundation, IRBLleida, Universitat de Lleida, Av. Alcalde Rovira Roure, 80, 25198 Lleida, Spain; 14 Public Health Agency of Catalonia, Health Department, Government of Catalonia, C. Roc Boronat 81-95, 08005 Barcelona, Spain; 15 Philip R. Lee Institute for Health Policy Studies, University of California San Francisco, 3333 California St., Ste. 265, San Francisco, CA 94118 USA

**Keywords:** Mental disorders, Clinical trial, Smoking cessation, Telephone, Quitlines

## Abstract

**Background:**

Up to 75% of inpatients with mental disorders smoke, and their life expectancy is decreased by up to 25 years compared to the general population. Hospitalized patients without monitoring after discharge quickly return to prehospitalization levels of tobacco use. The aim of the 061 QuitMental study is to assess the effectiveness of a multicomponent and motivational telephone-based intervention to stop smoking through a quitline addressed to smokers discharged from mental health hospital wards.

**Methods:**

A pragmatic randomized controlled trial, single blinded, will include 2:1 allocation to the intervention group (IG) and the control group (CG). The IG will receive telephone assistance to quit smoking (including psychological and psychoeducational support, and pharmacological treatment advice if required) proactively for 12 months, and the CG will receive only brief advice after discharge. The sample size, calculated with an expected difference of 15 points on smoking abstinence between groups (IG, 20% and CG, 5%), α = 0.05, β = 0.10, and 20% loss, will be 334 participants (IG) and 176 participants (CG). Participants are adult smokers discharged from psychiatric units of five acute hospitals.

Measurements include dependent variables (self-reported 7-day point prevalence smoking abstinence (carbon monoxide verified), duration of abstinence, number of quit attempts, motivation, and self-efficacy to quit) and independent variables (age, sex, and psychiatric diagnoses). In data analysis, IG and CG data will be compared at 48 h and 1, 6, and 12 months post discharge. Multivariate logistic regression (odds ratio; 95% confidence interval) of dependent variables adjusted for potential confounding variables will be performed. The number needed to treat to achieve one abstinence outcome will be calculated. We will compare the abstinence rate of enrolled patients between groups.

**Discussion:**

This trial evaluates an innovative format of a quitline for smokers with severe mental disorders regardless of their motivation to quit. If effective, the pragmatic nature of the study will permit transfer to routine clinical practice in the National Health System.

**Trial registration:**

ClinicalTrials.gov, NCT03230955. Registered on 24 July 2017.

**Electronic supplementary material:**

The online version of this article (10.1186/s13063-018-3106-5) contains supplementary material, which is available to authorized users.

## Key points

- This study is the first designed specifically for mental health patients with a long-term follow-up of 1 year, as other studies have performed follow-up periods of 6–7 months maximum.
- This trial evaluates an innovative quitline for smokers with severe mental disorders regardless of their motivation to quit.
- If effective, the pragmatic nature of the study will permit transfer to routine clinical practice in the National Health System.

## Background

Smoking prevalence has declined over the last decade in developed countries. However, in some vulnerable populations, such as people suffering from mental disorders, the prevalence has not followed this trend [1–3]. People with mental disorders are more likely to smoke, and the greater number of lifetime psychiatric diagnoses, the greater the probability of smoking [4]. In Catalonia, 74.4% of patients hospitalized for mental disorders smoke [5], which is three times higher than the smoking rate (26.9%) in the general population [6]. In addition, these patients start to smoke at an early age, experience higher levels of nicotine dependence, and have more difficulty quitting [7, 8].

The reasons for higher consumption of tobacco among people with mental disorders are not clear. Several explanations have been suggested, including genetic factors or/and a low socioeconomic level [9]. Another explanation sometimes invoked is the self-medication hypothesis, which suggests that patients with mental disorders, and particularly those with schizophrenic diagnoses, smoke to ameliorate their symptoms. However, this hypothesis is increasingly rejected [10, 11].

Tobacco is the cause of death for 48–53% of people with mental illness [12, 13] primarily for cardiovascular and respiratory diseases, cancer, and diabetes [14]. For people with severe mental illness, life expectancy is reduced up to 10–17 years, mainly for illnesses caused or worsened by smoking [15–17]. The persistent high prevalence of tobacco use within this population highlights how public health and healthcare services have neglected the needs of this disadvantaged population [18]. Historically, tobacco consumption has been considered a “minor problem” among patients with mental disorders; it was believed that these patients were not interested in quitting [19] and even that smoking helped patients control their disorder [20]. Some of these beliefs may be reflected in smoking cessation research. While there have been approximately 9000 smoking cessation trials among general population smokers, there have been only 20 such trials among patients with mental disorders [21]. The few studies that have been done, however, indicate that patients with mental disorders can achieve significant rates of abstinence [20].

Over the last decade, some jurisdictions have implemented cost-effective measures, as proposed by the World Health Organization (WHO), to curb smoking among persons with mental disorders [20]. These measures are mainly policies for smoke-free areas, treatment to quit smoking, and community measures of sustained support to quit [22].

Hospitalization in smoke-free psychiatric centers increases the quality of life in both patients and professionals, and decreases the exposure to second-hand smoke [23]. In addition, such policies increase quit attempts by patients and their expectations of staying abstinent [24, 25]. Only total smoke-free areas completely protect patients from second-hand smoke [26]. In Spain, the tobacco control Law 42/2010 bans smoking in both indoors and outdoors areas of acute hospitals including psychiatric units. This legal framework, which implies abstinence of patients during hospitalization, creates a unique opportunity to promote smoking cessation among patients with mental disorders.

Concerning treatment to quit smoking, the use of self-help materials, telephone advice, and psychological and pharmacological treatments have been shown to be effective [27, 28]. Although interventions to promote smoking cessation among patients with mental disorders aim to achieve abstinence, it is often necessary to first promote motivation to quit [27]. Thus, these patients would be able to make serious attempts to quit smoking when hospitals have trained professionals to respond to their needs and offer them pharmacological and psychological aids [29]. Interventions that combine pharmacological treatment and psychological therapy—primarily cognitive-behavioral psychological treatment—increase the likelihood of tobacco cessation in patients with mental disorders [30–32]. However, without patient monitoring after discharge, smoking is restored to previous levels in 2 weeks [33, 34]. Only a few clinical trials [21, 35, 36] have demonstrated the efficacy of multicomponent interventions (which included motivational, psychosocial, and pharmacological components) addressed to patients with mental disorders after discharge. In these studies, the intervention group showed higher abstinence rates, more quit attempts, and fewer cigarettes per day as compared to controls [21, 35, 36]. In addition, as shown in the general population, it is likely that patients with a mental disorder who quit smoking also reduce their levels of anxiety, increase their quality of life, and obtain better results in abstinence from other drugs [37].

In relation to community measures, “quitlines” (i.e., telephone counseling) stand out as an effective support in the general population [38]. However, this resource has so far been little used among populations with mental disorders [39]. The few studies conducted with mentally ill patients showed that a quitline intervention combined with nicotine replacement therapy (NRT) slightly but significantly increased 7-day point prevalence abstinence at 6 months follow-up, reduced the number of cigarettes consumed per day, and increased readiness to quit smoking [36, 39].

In Catalonia, the health information and care telephone line “061 CatSalut Respon” provides a free support program to quit smoking, which acts as a quitline. A nursing team, experts in telephone assistance and smoking cessation, provide an individualized intervention to quit smoking through intensive care for 1 year. However, this service has not so far been used for patients with mental disorders. Quitlines may have some advantages over other cessation services, as they can increase access, are less costly than face-to-face visits [38], and offer a solution to the lack of systematic interventions in patients with mental disorders especially after discharge [34, 40]. However, contact with the quitline is usually initiated by the smoker, and is not usually initiated proactively by the treatment provider. Therefore, we propose a pragmatic randomized controlled trial that uses the existing resources of 061 CatSalut Respon to engage patients with mental disorders identified during hospitalization in acute psychiatric hospital units.

The aim of this study is to assess the effectiveness of a telephone-based (quitline) intervention for smoking cessation addressed to smokers with severe mental disorders after discharge from adult inpatient acute units (the 061 QuitMental study). The intervention group (IG) will receive a telephone intervention to quit smoking (including psychological and psychoeducational support and pharmacological treatment advice, if required) proactively for 12 months, and the control group (CG) will receive only brief counseling after discharge.

The specific objectives of the 061 QuitMental study are as follows:

- to assess the effectiveness of the intervention in relation to the characteristics of the smokers (mental disorder, comorbidity, age, sex, and educational level and occupation);
- to compare the levels of motivation to quit smoking, self-efficacy, attempts to quit smoking, and the likelihood of smoking abstinence in the intervention group and the control group;
- to describe the use and satisfaction with the quitline in the intervention group and the control group; and
- to assess the feasibility of this intervention to systematically offer it at a community level.

**Fig. 1:**
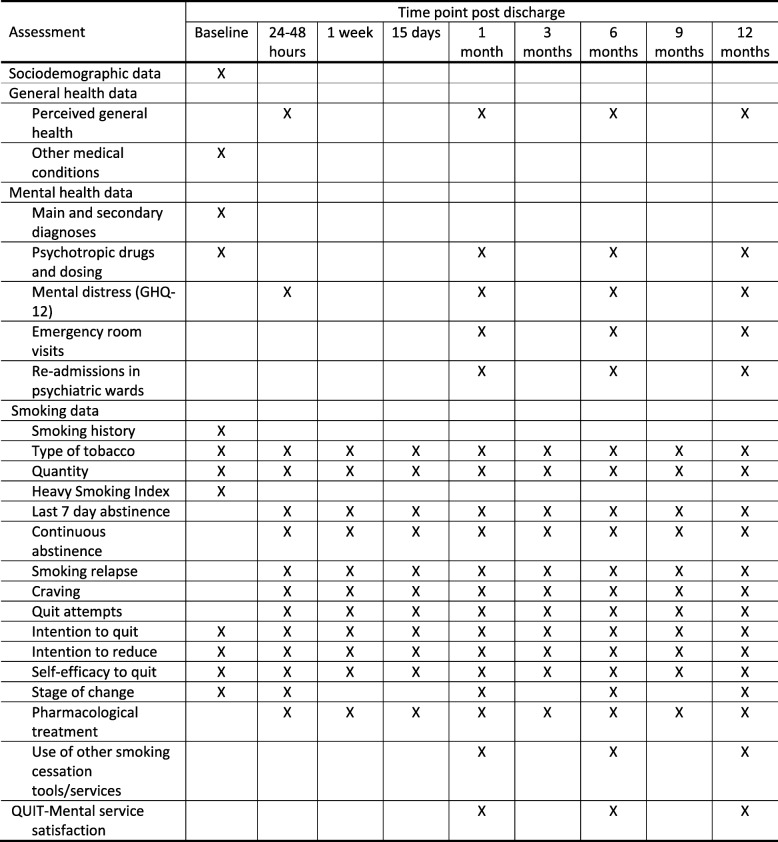
Study variables and collection time points. GHQ-12 12-item version of the General Health Questionnaire

These objectives will be analyzed by comparing the results obtained from the IG and the CG at several time points post discharge: 48 h and 1, 6, and 12 months (Fig. 1).

The hypotheses of this study are the following:

- IG participants, as compared to CG participants, will demonstrate higher rates of biochemically verified 7-day point prevalence smoking abstinence, at all evaluation time points (48 h and 1, 6, and 12 months post discharge).
- IG participants, as compared to CG participants, will experience longer periods of sustained abstinence as demonstrated by sequential biochemically verified 7-day point prevalence smoking abstinence, at all evaluation time points.
- IG participants who continue to smoke, as compared to CG participants, will show greater reduction in the number of cigarettes smoked per day, at all evaluation time points.
- IG participants who continue to smoke, as compared to CG participants, will show an increase in the progression of their readiness to quit smoking, based on stages of change, at all evaluation time points.
- IG participants, as compared to CG participants, will have a higher level of self-efficacy to quit at all evaluation time points.

## Methods

This project arises from the collaboration between the Catalan Institute of Oncology, the Public Health Agency of Catalonia, and the 061 CatSalut Respon program, which is a public telephone service from the National Health System about health topics addressed to all citizens.

### Design

This study is a pragmatic randomized controlled trial (RCT) with two groups: intervention group (*n* = 334) and control group (*n* = 176). The subjects will be followed up to 12 months. The duration of the study is 3 years and is divided into three phases: phase I, intervention design and pilot study for 1 month; phase II, recruitment during 24 months; and phase III, analyses of the effectiveness of the intervention, publication of the results, and dissemination (6 months after phase II).

### Study population

The study population includes smokers with mental disorders admitted to the mental health wards of five acute care hospitals of Barcelona (Hospital Clinic i Provincial de Barcelona, Hospital de la Santa Creu i Sant Pau, Hospital Universitari Vall d’Hebron, Hospital Universitari de Bellvitge, and Hospital Hestia Duran i Reynals). The inclusion criteria are: patients who consider themselves smokers, both daily and occasional smokers (less than 1 cigarette per day); adults of both sexes aged 18–76 years; patients who have stayed in an acute or detoxification mental health unit for more than 24 h; patients with a telephone, fixed or mobile; residents in the metropolitan area of Barcelona; and patients who provide informed consent. The exclusion criteria are: patients discharged from the psychiatric emergency rooms; patients with dementia or brain damage; patients who do not speak or read Spanish or Catalan; pregnant women; patients with hearing and/or speech deficit; patients who are trying to quit smoking in another center or using another intervention; patients who have voluntarily requested discharge; patients who are discharged to another inpatient unit; and patients who plan to move their household outside the Barcelona metropolitan area within the next 24 months.

Clinical professionals (coordinated by a researcher from each hospital) will evaluate whether patients met the inclusion/exclusion criteria.

### Sample size and randomization

Data from the five centers indicate that each year 1744 patients are admitted to their acute and detoxification mental health units, of which 70% are expected to be smokers [5]. Setting a risk of α = 0.05 and β = 0.10, and with 2:1 randomization, 334 subjects are required in the IG arm and 167 in the CG arm to detect a statistically significant difference of 15 percentage points between the proportion of abstinence in both groups (IG abstinence rate, 20% and CG abstinence rate, 5%) after 12 months, with an estimated 20% loss to follow-up. Hence, participants will be randomized into the IG and the CG with 2:1 allocation using mapping software. The main reason for the unequal allocation (2 IG:1 CG) was ethical, in order to maximize the number of participants included in the treatment group, as the treatment is supposed to be beneficial for them.

### Variables

#### Outcome measures

##### Primary outcome measure

Self-reported 7-day point prevalence smoking abstinence, biochemically verified with expired carbon monoxide (CO) measures, is the primary outcome. Seven days of abstinence has been chosen as a consumption measure [41]. This variable will be recorded in every call of the intervention (48 h, 1 week, 15 days, and 1, 3, 6, and 12 months post discharge). Abstinent subjects at 1, 6, and 12 months in either of both groups (IG or CG) will be invited to attend a consultation at their hospital to verify their abstinence through the detection of exhaled carbon monoxide. The abstinence rate between the IG and the CG will be compared at 1, 6, and 12 months. In each call, nicotine withdrawal symptoms will be assessed according to the scale proposed by Hughes and Hatsukami [42].

##### Secondary outcome measure

We will also record other smoking variables such as continuous abstinence. Moreover, for nonabstinent patients we will ask about their tobacco use (quantity), type of tobacco consumed (cigarette/cigar/other), number of quit attempts, and other variables included in the “smoking” section in Fig. 1. Moreover, using an ad-hoc Likert scale from 0 to 10 we will evaluate the level of self-efficacy to quit or reduce consumption, the intention to quit, and the intention to reduce consumption. The individual’s willingness to quit will be also evaluated according to the Prochaska and Di Clemente Stages of Change model, which measures four stages of readiness to quit smoking (precontemplation, contemplation, action, and maintenance) [43].

#### Independent variables

Using a data collection form we will collect the following information:

- Sociodemographic data such as sex, age, occupation, and highest level of education attained.
- History and pattern of tobacco use, which will include information to obtain the Heavy Smoking Index as a measure of nicotine dependence self-reported by the participants [44, 45].
- General health data, such as self-perceived general health assessment by the question “how would you describe your health in a general way?”, and other medical conditions.
- Mental health data, such as diagnoses according to the DSM-5 groups of diagnostic criteria [46] and psychotropic treatment, will be obtained from participants’ clinical records by the researchers, whereas information about the number of emergency room visits, readmissions to psychiatric wards, and evaluation of mental distress measured using the 12-item version of the General Health Questionnaire (GHQ-12) [47, 48] will be self-reported by the participants.
- Variables of smoking cessation services reported by the participants, such as, if used, any other strategy for tobacco cessation, will be registered. It will also be recorded whether the patient has actively spoken with their psychiatrist, psychologist, nurse, or family doctor about their participation in the quitline, whether the professionals have agreed, and whether they have helped them, to assess the possible influence of these variables on the success (or failure) of the intervention.

Collected variables and their collection frequency are detailed in Fig. 1.

### Procedure

The research team designed a telephone-based intervention with a proactive approach to help patients quit smoking after discharge from hospitals. The intervention will take into account the recommendations made by a group of experts in the design of telephone-based interventions (quitlines) aimed at patients with mental illnesses [39]. The intervention will also be based on the protocol of the general quitline of 061 CatSalut Respon and a clinical intervention guide for smoking cessation in patients with mental disorders [28]. Depending on the case, the intervention will be directed to: increase motivation to quit; achieve abstinence; prevent relapses if achieving abstinence; reduce consumption and increase motivation to quit smoking when the patient is still not ready or motivated to quit; and/or recommend pharmacological treatment.

The intervention will be conducted by 061 CatSalut Respon nurses within the first 48 h, 1 week, 15 days, and 1, 3, 6, 9, and 12 months after discharge. IG subjects will be contacted by the trained nurses and will provide an intervention strategy adapted to each patient situation in each call (whether they smoke, take pharmacological treatment to quit, are ready for a quitting day calls, “D day”, etc.) according to an algorithm procedure (Fig. 1). The first telephone call at 48 h will serve as a starting point for the quitline intervention outside the hospital and to take baseline data post discharge.

CG subjects will be contacted after the first 48 h following discharge by nonclinical telephone assistants who will provide brief advice (only in the first call) and collect data for comparison, at this time (48 h), 1, 6, and 12 months post discharge. The investigators trained the nonclinical telephone assistants in order to conduct the interviews in a neutral tone following the wording of each of the questions and not giving health recommendations, as they are not qualified to do so. For patients who express their desire to quit, telephone assistants suggest they talk with their outpatient clinic doctor, nurse, or psychologist to receive aid*.*

**Fig. 2:**
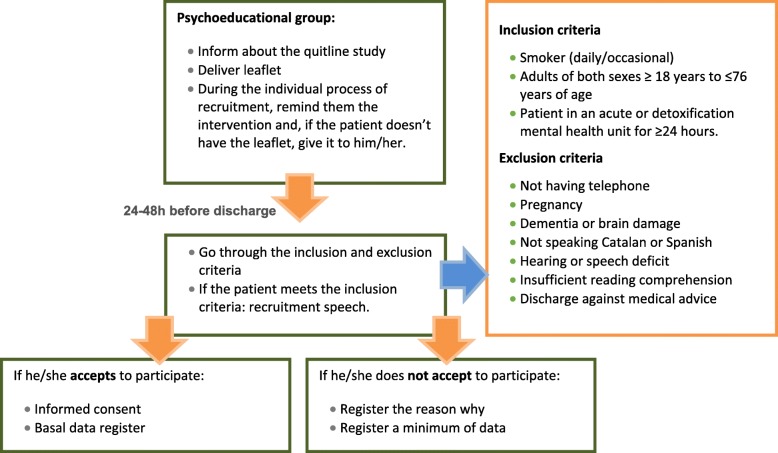
Recruiting process

For those patients who met the criteria, clinicians will invite them to participate and will deliver an informative leaflet of the study (Additional file 1). Informed consent will be requested within 24 h before discharge (see Fig. 2, recruiting process). Clinicians will register participants’ data in a software program that will be used for randomization and to transfer this information to 061 CatSalut Respon to contact the participants. All of this baseline information is registered in the software during the hospital stay. Finally, on the day of discharge the subjects are automatically assigned randomly to one of the two groups (IG or CG) by the software. The study is blinded to prevent professionals from the hospital, and patients, from knowing to which group they have been assigned.

### Telephone-based intervention components

The intervention is based on cognitive-behavioral therapy. The intervention will include components based on Bandura’s social learning theory, social cognitive theory [49], and the transtheoretical model of change [43]. This theory allows evaluating the patient motivation phase and adapting the interventions according to each phase [27].

These components are included in the training curriculum aimed at 15 nurses from 061 CatSalut Respon who will deal with patients with mental disorders from the IG. The specific training will include the definition and description of the most common mental disorders, symptoms, usual treatments, warning signs, and management, as well as the peculiarities of the smoking cessation treatment in these patients (Table 1).

**Table 1:** Components of the formative curriculum

• Mental disorders, symptomatology, and general treatment	
• Content, extension, and frequency of the proactive calls	
• Withdrawal syndrome assessment in the mental health context	
• Pharmacological recommendations about nicotine replacement therapy	
• Peculiarities of smoking cessation treatment in patients with mental disorders, alert signs, and management	
• Motivational intervention	
• Communication strategies	

**Fig. 3:**
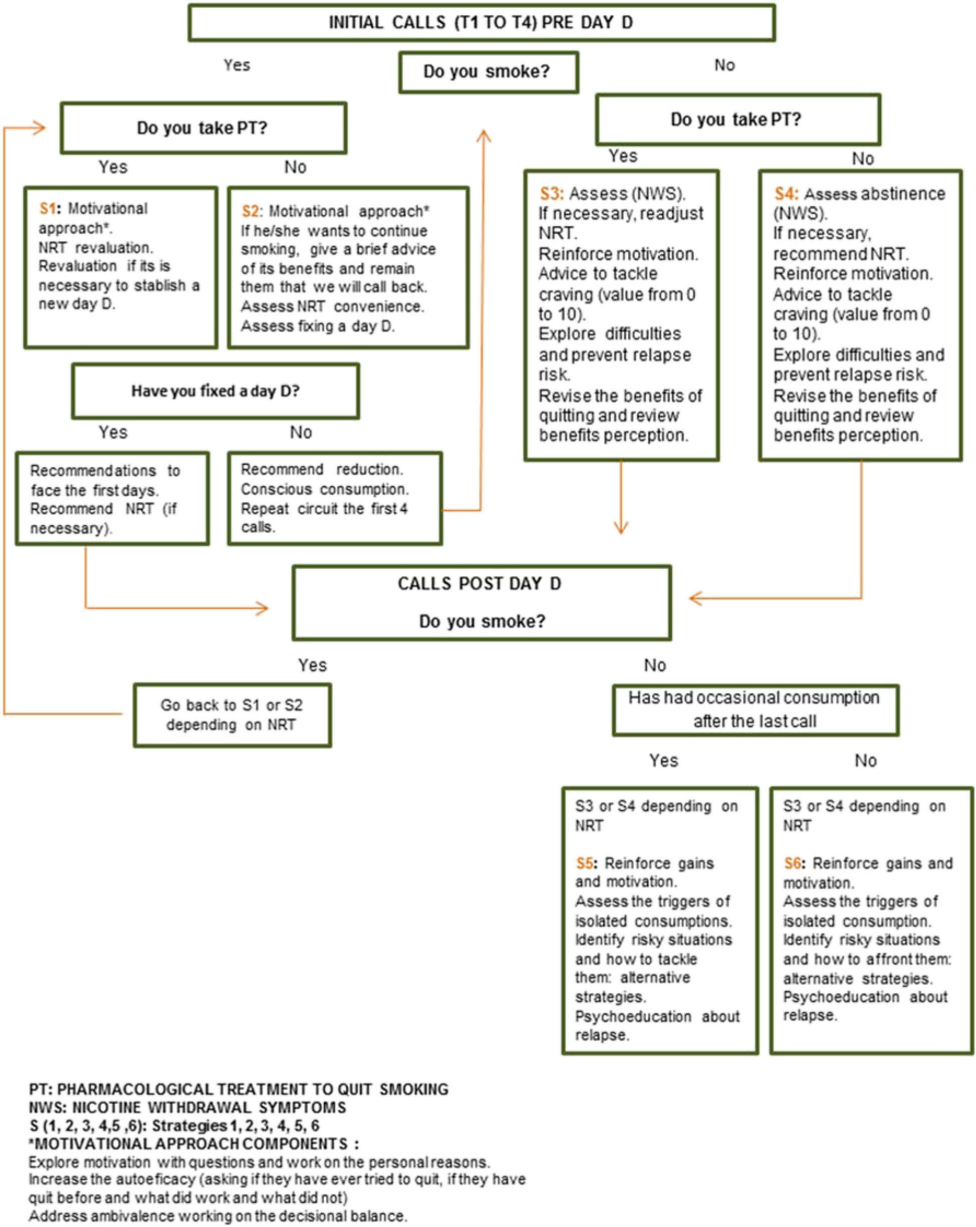
Strategy intervention in the intervention group. NRT nicotine replacement therapy

Trained nurses will help those patients to identify their barriers and opportunities to achieve abstinence. They will also help them to set objectives, strategies to tackle “craving”, positive reinforcement toward progress, and increase their self-efficacy. If necessary, nurses will recommend NRT according to the protocol of a clinical guideline [28], as it is a medication that does not require medical prescription in Spain. Nurses will have a written protocol and an algorithm to deliver the intervention strategy according to each patient’s situation (Fig. 3). Nurses will recommend the patients inform their psychiatrist and/or psychologist that they are receiving this telephone-based intervention to quit smoking.

All patients in the intervention group will receive eight telephone calls during 1 year (48 h, 1 week, 15 days, and 1 , 3 , 6 , 9, and 12 months post discharge), regardless of whether they continue smoking or not. The intervention lasts for 12 months, unless the patient rejects to continue participating in the study. The intervention adapts its objectives depending on the smoking status of the patient in each call (see Fig. 3 in which the different strategies are explained according to the smoking status assessed in each call, whether he/she is abstinent or not, is willing to allocate a “D day”, takes NRT).

### Data collection

A computer-specific application for the study will be designed for data recording and management, including randomization. The software will be created following the current standards of security, privacy, and confidentiality. All study researchers will have access to the application using a personal username and password. Clinicians from each center will only be able to register and have access to review information from patients from their own center. The 061 CatSalut Respon nurses will obtain data from patients registered in the five centers to do the telephone intervention. Collected data will be exclusively used to carry out the intervention (IG or CG), supervision, and evaluation. Patients will be informed about the application uses and purposes, the existence of their profile including their personal details, and the person responsible for their treatment, along with their access rights, rectification, cancellation, and opposition. Once the patient has given his/her consent, clinicians will input data from the basal interview into the application.

Once the study is completed, professionals from the hospitals and 061 CatSalut Respon will no longer have access to the database. The ICO is responsible for properly saving the files and its protection by encrypted secure systems, until its eventual destruction (5 years after study completion). The database will not identify patients in any way, creating an individual encrypted code. Data collected during the study will be included in a file registered by the Spanish Data Protection Agency.

The intervention and the computer application will be firstly piloted in the five participating centers for 1 month for its evaluation before starting the study, to assess the adequacy of the fieldwork circuit and make minor changes in the protocol, if necessary.

### Data analysis

The abstinence prevalence will be analyzed by groups taking into account sociodemographic determinants. Logistic regression analysis will be used to assess the effectiveness of the intervention (OR and 95% confidence interval) with regard to the CG, using the different dependent variables previously described (abstinence, reduction of the number of cigarettes, motivation, etc.). Thus, the IG and the CG will be compared in different moments of the follow-up. In addition to randomization, to warrant comparability, we will fit multivariate logistic models to adjust for potential confounding variables. All analyses will be carried out by “intention to treat”, to take into account possible losses during follow-up. We will also analyze resource use, such as the number of calls, satisfaction with the quitline, and type of treatment offered, among others.

### Ethical considerations

The intervention protocol has been approved by the Ethics Committee for Clinical Research (CEIC) from the Bellvitge Biomedical Research Institute (IDIBELL) (reference: PR276/16), as well as by the Ethics Committee of each of the five participating hospitals. The study protocol has been registered under ClinicalTrials.gov (NCT03230955, approved 24 July 2017).

At all times, requirements established by Law 14/2007, of 3rd July, on Biomedical Research will be met by researchers. The study will take place in compliance with the Declaration of Helsinki.

Smokers who meet the inclusion criteria will receive a brief explanation of the purpose of the study. They will also be given an information sheet and they will be asked to fill in the informed consent. The information will be protected according to the standards established by Spanish Law 15/1999 on the Protection of Personal Data.

The study termination criteria are the following (the first to be met): conclusion of the study procedures; if a patient explicitly states that he/she do not want to continue to participate in the study; and if a patient has repeatedly not been located in each of the off-tracking visits from both the intervention or control group during the 12 months of tracking.

## Discussion

Smoking has serious implications for people with severe mental illnesses, mainly in terms of quality of life and mortality but also in economic costs and marginalization. Hospital mental health units are an optimal setting to intervene and promote patient engagement in tobacco cessation treatment [21]. For this reason, it is necessary to offer and improve a variety of interventions to motivate and promote cessation among these patients.

Patients with mental health disorders are usually excluded from smoking cessation trials [20], and consequently there is scarce evidence for the effectiveness of quitlines in this population. As these patients usually have high comorbidity and high smoking dependence, they represent a complex population for smoking cessation intervention. They need more intensive counseling, pharmacological intervention, and longer follow-up support to achieve cessation [20], so there is a need to tailor these strategies to this population [50]. Only a few studies of quitline effectiveness have included mental health patients and, usually, quitline interventions have not been tailored to this population [51]. This is the first randomized trial to test a quitline intervention specifically tailored to the needs of mental health patients that incorporates a long-term follow-up of 1 year, since other studies have performed follow-up periods from 6 to 7 months [52] and with smaller samples [53].

The large sample, together with the long follow-up period, will allow us to perform other subgroup analyses in addition to assessing smoking cessation. The main expected outcome is an increased rate of CO-verified 7-day point prevalence abstinence in the intervention group, as compared with the control group. All smokers, regardless of their level of motivation to quit, will be eligible to participate. Analyses will consider covariates such as psychiatric diagnoses, use of other psychoactive substances, psychological distress, mental illness relapse and rehospitalization, and sociodemographic variables. Other relevant outcomes for comparison will include the level of motivation to quit, number of quit attempts, and level of psychological distress.

There are several potential limitations. In Catalonia, Spain, patients who have high motivation to quit smoking at the time of discharge from acute mental health hospitals are referred to a face-to-face intensive smoking cessation intervention with free pharmacotherapy for smoking cessation. These patients are excluded from our study and, therefore, our sample does not include patients with high motivation to quit smoking. Nonetheless, we will assess all patients who meet the study inclusion criteria and compare demographic, psychiatric, and tobacco use characteristics of those who accept or decline enrollment. Another limitation, derived from the long-term follow up design, is loss to follow-up that could bias follow-up findings. We will perform an attrition analysis comparing characteristics of those followed to those not followed at each time point. In addition, to preserve statistical power needed to observe a 15% difference in the quit rate between conditions, we have increased the baseline sample size to accommodate an anticipated 20% loss to follow-up at 12 months. To increase follow-up data collection, clinicians will collect contact telephone numbers for the participant and at least two family members or caregivers. There is potential for underreporting of smoking status at follow-up. Participants reporting nonsmoking status at follow-up receive a reminder and a clinic appointment to complete exhaled CO testing.

If effective, this intervention could be included in the Catalan Health System and regularly offered to mental health patients who smoke, no matter their motivation to quit. We need to offer a wide range of possibilities to intervene in smoking cessation in this population as their smoking prevalence has not decreased in the last decade (Additional file 2).

## Additional files

Additional file 1: Informative leaflet for the quitline (DOCX 529 kb)

Additional file 2: SPIRIT 2013 checklist: recommended items to address in a clinical trial protocol and related documents (DOC 120 kb)
