# Supplementary material for: Effectiveness of a telephone-based intervention for smoking cessation in patients with severe mental disorders: study protocol for a randomized controlled trial
Source: Trials. 2019 Jan 11;20:38. doi: 10.1186/s13063-018-3106-5 (PMC6329054; doi:10.1186/s13063-018-3106-5)
Supplement: Supplementary file 1 — Informative leaflet for the quitline (DOCX 529 kb) [file 13063_2018_3106_MOESM1_ESM.docx]

**Annex 1:** Informative leaflet of the quitline
